# Sand fly blood meal volumes and their relation to female body weight under experimental conditions

**DOI:** 10.1186/s13071-024-06418-y

**Published:** 2024-08-23

**Authors:** Věra Volfová, Magdalena Jančářová, Petr Volf

**Affiliations:** Department of Parasitology, Faculty of Science, Charles University, Prague 2, CZ 128 43 Czech Republic

**Keywords:** *Phlebotomus*, *Lutzomyia*, *Sergentomyia*, Blood meal, Prediuresis, Haemoglobin

## Abstract

**Background:**

Sand fly females require a blood meal to develop eggs. The size of the blood meal is crucial for fecundity and affects the dose of pathogens acquired by females when feeding on infected hosts or during experimental membrane-feeding.

**Methods:**

Under standard laboratory conditions, we compared blood meal volumes taken by females of ten sand fly species from four genera: *Phlebotomus*, *Lutzomyia*, *Migonomyia*, and *Sergentomyia*. The amount of ingested blood was determined using a haemoglobin assay. Additionally, we weighed unfed sand flies to calculate the ratio between body weight and blood meal weight.

**Results:**

The mean blood meal volume ingested by sand fly females ranged from 0.47 to 1.01 µl. Five species, *Phlebotomus papatasi*, *P. duboscqi*, *Lutzomyia longipalpis*, *Sergentomyia minuta*, and *S. schwetzi*, consumed about double the blood meal size compared to *Migonomyia migonei*. The mean body weight of females ranged from 0.183 mg in *S. minuta* to 0.369 mg in *P. duboscqi*. In males, the mean body weight ranged from 0.106 mg in *M. migonei* to 0.242 mg in *P. duboscqi*. Males were always lighter than females, with the male-to-female weight ratio ranging from 75% (in *Phlebotomus argentipes*) to 52% (in *Phlebotomus tobbi*).

**Conclusions:**

Females of most species took a blood meal 2.25–3.05 times their body weight. Notably, the relatively tiny females of *P. argentipes* consumed blood meals 3.34 times their body weight. The highest (Mbl/Mf) ratios were found in both *Sergentomyia* species studied; females of *S. minuta* and *S. schwetzi* took blood meals 4.5–5 times their body weight. This parameter is substantially higher than that reported for mosquitoes and biting midges.

**Graphical Abstract:**

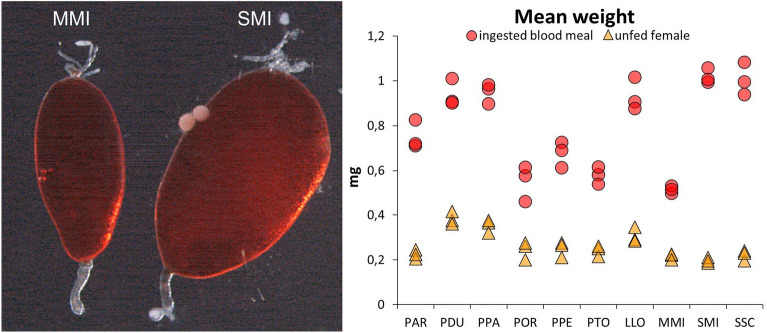

**Supplementary Information:**

The online version contains supplementary material available at 10.1186/s13071-024-06418-y.

## Background

Due to their haematophagous behaviour, phlebotomine sand flies (Diptera: Psychodidae, Phlebotominae) are key vectors in the transmission of medically and veterinary important pathogens, including various *Leishmania* species, *Bartonella bacilliformis*, and several phleboviruses such as the Toscana virus [1, 2].

Sand fly females require a blood meal to obtain the necessary nutrition for successful reproduction. Feeding behaviour and the size of the ingested blood meal can vary depending on the vertebrate source [3, 4]. Under laboratory conditions, host choice is usually limited to commonly used laboratory animals such as mice, hamsters, and rabbits [5, 6]. Alternative blood-feeding on artificial membrane feeders often results in lower feeding success compared to feeding on live animals [7, 8]. Determining the mean blood volume ingested by a single sand fly female is crucial for specifying the maximum tolerable number of females per host in a feeding trial. Additionally, the volume of the blood meal is essential for calculating infective doses in laboratory experiments as it affects the number of infective agents taken up during xenodiagnoses or membrane feeding in vector competence studies [9]. For example, in low-susceptible sand fly species the *Leishmania* infections are dose dependent but in highly susceptible ones, like *Phlebotomus argentipes* and *P. orientalis*, even 1–2 *Leishmania donovani* parasites are enough to initiate mature infections. This low infection dose corresponds to 2 × 10^3^ parasites/ml [7].

Previous studies, often focussing on a single sand fly species, have reported blood meal sizes ranging from 0.4 to > 1.0 µl of ingested blood [3, 7, 10–15]. However, the variety of methods used makes precise interspecific comparison difficult. Notably, data obtained by gravimetry may be underestimated as this method omits the ability of blood-feeding Nematocera to excrete excess water and concentrate the blood meal during feeding [16, 17]. This process, known as prediuresis, has been described in various sand fly species [3, 10, 18, 19]. Therefore, methods based on haemoglobin or protein estimation provide more accurate and reproducible results of the total amount ingested.

In other haematophagous Nematocera, particularly in *Aedes* and *Anopheles* mosquitoes, a positive correlation between blood meal volume and female body size has been documented [20, 21]. However, extensive prediuretic excretion allows some small-sized species to significantly increase the amount of ingested blood [16]. In sand flies, previous studies [7, 15] suggested that the blood meal volume does not interspecifically correlate with body size, but this relationship has not been closely examined. Similarly, data on sand fly body weight are scarce [10, 22].

In this study, we used ten laboratory-reared sand fly species and haemoglobinometry to determine the amount of ingested blood under standard conditions, which are the same conditions used for experimental infections with *Leishmania* or phleboviruses. Additionally, we compared the body weight of sand fly females and males and analysed the relationship between blood meal size and female body weight.

## Methods

### Sand fly colonies and their maintenance

Ten well-established colonies of ten species from four sand fly genera including three *Phlebotomus* subgenera were used: *Phlebotomus (Euphlebotomus) argentipes* Annandale & Brunetti 1908; *P. (Phlebotomus) duboscqi* Neveu-Lamaire, 1908; *P. (Larroussius) orientalis* (Parrot, 1936); *P. (Larroussius) perniciosus* Newstead, 1911; *P. (Phlebotomus) papatasi* (Scopoli, 1786); *P. (Larroussius) tobbi* Adler, Theodor & Lourie, 1930; *Lutzomyia longipalpis* (Lutz & Neiva, 1912); *Migonemyia migonei* (França 1920); *Sergentomyia minuta (*Rondani, 1843) and *S. schwetzi* (Adler, Theodor and Parrot, 1929). The colonies were maintained in the insectary of the Department of Parasitology, Charles University, in Prague, under standard conditions (at 26 °C, 60–70% humidity, and 14 h light/10 h dark photoperiod) as described previously [5]. Adults were offered 50% sucrose ad libitum. Sand fly females were fed on either anaesthetized BALB/c mice (*P. argentipes, P. duboscqi*, *P. papatasi, L. longipalpis*, *S. schwetzi*) or mechanically restrained New Zealand White (NWZ) rabbits (*P. orientalis*, *P. perniciosus*, *P. tobbi*, *M. migonei*) or leopard geckos (*S. minuta*). The blood-feeding was routinely carried out on the animal host for about 60 min in most colonies. *Sergentomyia minuta* females need more time for full engorgement [15]; thus, they were allowed to feed on reptiles for 2 h.

### Animal maintenance

BALB/c mice originating from AnLab s.r.o. (Harlan Laboratories, USA) were maintained in T3 breeding containers (Velaz) equipped with bedding (German Horse Span, Pferde a.s.) and breeding material (Woodwool) and provided with a standard feed mixture (ST-1, Velaz) and water ad libitum, with a 12 h light/12 h dark photoperiod, at 22–25 °C and 40–60% humidity. NZW rabbits (originating from AnLab s.r.o.) were kept in breeding boxes (Velaz) equipped according to guidelines and legislation, provided with a standard feeding mixture for rabbits (Biopharm), hay, and water ad libitum as described in Ticha et al. [15]. Leopard geckos, *Eublepharis macularius* (Blyth, 1854), were kept in glass terraria (60 × 40 × 35 cm) at 24 °C and 32 °C in a basking area, 12/12 light/dark regime and continual access to water. Their feeding was performed three times a week with crickets. The exposure to sand flies was performed once every 3 weeks to allow the animal host to recover.

### Body weight of sand flies

While body size of sand flies has been mostly determined using morphometric parameters to date, in this study the body weight of sand fly females was correlated with the weight of ingested blood. Newly emerged adults were released from rearing pots into nylon cages (40 × 40 × 40 cm) with wet cotton wool on the top of the cage to supply sufficient humidity. The adults from at least four pots were used per trial. No sugar meal was provided for 24 h. Then, unfed sand flies were collected, immobilized on ice, carefully transferred into 0.5-ml micro test tubes (Eppendorf^®^), and weighed in batches of 20 flies on the Ohaus^®^ PR 124/E analytical balance (OHAUS Corp., USA). The measurement was repeated in six independent trials for both males and females of all studied sand fly species. The last measurement was done 12 months after the first one. The mean individual male (M_m_) and female (M_f_) body weights together with the M_m_/M_f_ ratio were calculated and statistically compared.

### Haemoglobin assay for measuring the blood meal volume

Haemoglobinometry was used to compare the blood meal size in ten sand fly species; the method chosen is independent of prediuresis and diuresis and provides precise assessment of the blood meal volumes ingested by sand fly females [7]. We used a modification with a commercially available kit, as described previously by Ticha et al. [15]. Sand fly females (100 per trial, 5–7 days old) were offered a blood meal on a routinely used animal host (same conditions as for maintenance of the colony). One hour post blood meal, fully engorged females were selected and immobilized on ice. Individual guts without Malpighian tubules were dissected in 20-mM TRIS-NaCl under a stereo microscope (Olympus SZH Stereo Microscope), transferred to microtubes with 1 ml dH_2_O, and stored in batches of ten guts per sample at − 70 °C. Sample homogenates were prepared by thorough mechanical homogenization. Afterwards, haemoglobin content was measured using Haemoglobin Assay Kit (MAK115, Sigma-Aldrich) following the manufacturer’s instruction in 96-well plates. The assay was calibrated by diluted calibrator provided in the kit (equivalent to 1 mg/ml haemoglobin). Fifty μicrolitres of the sample homogenate was loaded per well in quadruplicate, mixed with 200 μl reagent, and incubated 5 min at room temperature, and the absorbance was measured at 400 nm by Tecan-Infinite M 200 Fluorometer (Schoeller Instruments). The measurement was performed in three independent trials for each sand fly species in the study. The resulting haemoglobin content was compared to the haemoglobin concentration measured in the host blood (the same animal host individuals as used for experimental feeding) to determine a mean blood meal volume per female (V_bl_). In addition, mean blood meal mass (M_bl_) was related to the estimated mean weight of the unfed females (M_f_) in the experimental cohort.

### Statistical analysis

Statistical analysis was performed using standard Excel programme tests for Windows 10 (Microsoft^®^ Corp., USA) and Real Statistics Resource Pack software (Release 8.8.2) http://www.real-statistics.com. Comparisons of body weights and blood meal volumes in ten sand fly species were analysed using one-way analysis of variance (ANOVA) followed by Tukey-Kramer multiple comparison tests. The intraspecific differences in male and female body weights were tested by Student’s t-test. Shapiro-Wilk tests were used to analyse data for normality and Levene’s tests for homogeneity of variances. *P*-values < 0.05 were considered statistically significant.

## Results

### Comparison of blood meal volumes ingested by ten sand fly species

**Fig. 1:**
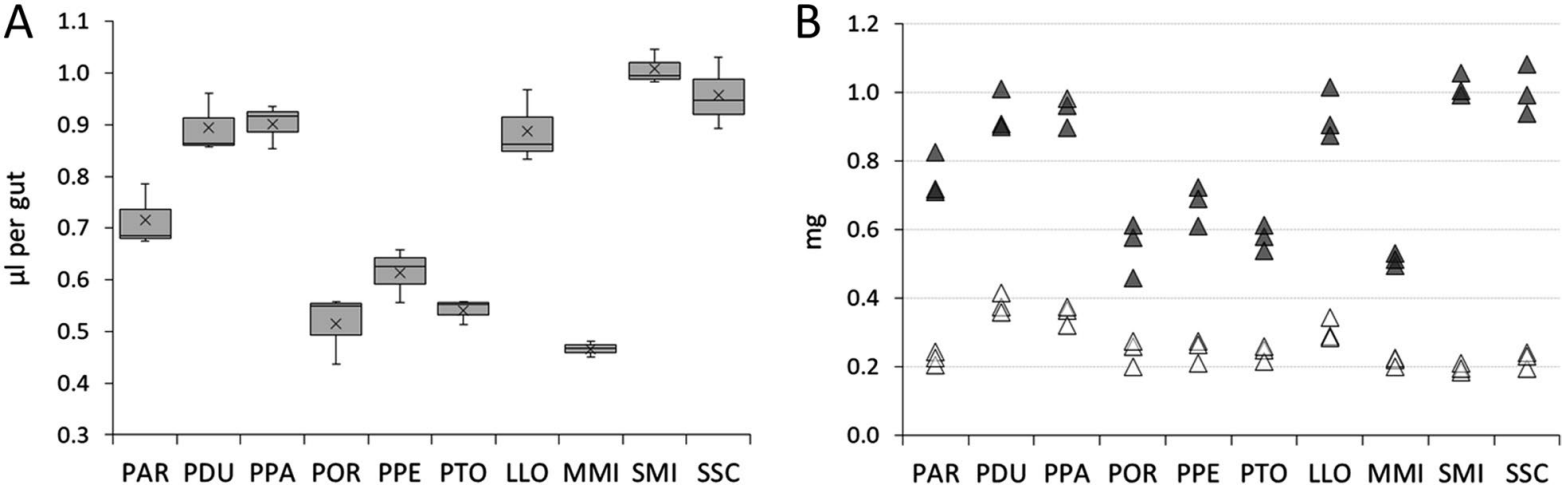
Blood meal size ingested by females of ten sand fly species. Volume of ingested blood meal (**A**) and comparison of blood meal weight with the mean body Δ weight of unfed females (**B**). Ten sand fly species were studied: *Phlebotomus argentipes* (PAR); *P. duboscqi* (PDU); *P. papatasi* (PPA); *P. orientalis* (POR); *P. perniciosus* (PPE); *P. tobbi* (PTO); *Lutzomyia longipalpis* (LLO); *Migonemyia migonei* (MMI); *Sergentomyia minuta (*SMI) and *S. schwetzi* (SSC). The columns represent an average from three independent samples (each sample comprising 10 sand fly specimens). The interspecific differences analysed by one-way ANOVA were highly significant in all studied parameters: the blood meal size (*F*_*(9,20)*_ = 44.16; *P* < 0.0001), unfed body weights (*F*_*(9,20)*_ = 15.87; *P* < 0.0001), and the M_bl_/M_f_ ratios (*F*_*(9,20)*_ = 150.94; *P* < 0.0001)

The size of ingested blood meal was determined in ten sand fly species using haemoglobin measurement. Significant interspecific differences were found; mean blood meal volume ranged from 0.47 µl in *Migonomyia migonei* to 1.01 µl in *Sergentomyia minuta* (Fig. 1A)*.*

Both *Sergentomyia* species took relatively big blood meals (V_bl_ 0.96 ± 0.07 µl and 1.01 ± 0.03 µl for *S. schwetzi* and *S. minuta*, respectively). Within *Phlebotomus*, similarities occurred between members of the same subgenus. *Phlebotomus duboscqi* and *P. papatasi* (both belonging to subgenus *Phlebotomus*) took very similar blood meal volume (V_bl_ 0.89 ± 0.06 µl and 0.90 ± 0.04 µl, respectively). Analogously, no statistical difference was not found in blood meal volumes between *Larroussius* species, *P. perniciosus*, *P. orientalis*, and *P. tobbi* (V_bl_ 0.61 ± 0.05 µl, 0.51 ± 0.07 µl, and 0.54 ± 0.03 µl, respectively). In *L. longipalpis*, the blood meal volume (V_bl_ 0.89 ± 0.07 µl) was very similar to those in *P. papatasi* and *P. duboscqi* (Fig. 1A). By contrast, the blood meal volume taken by *M. migonei* females was the lowest among all sand fly species tested (V_bl_ 0.47 ± 0.02 µl).

Data are vizualized in Fig. 1A. For the Tukey-Kramer multiple comparison test table, see Supplementary information (Additional file 1: Table S1).

### Body weight of sand fly females and males

**Fig. 2:**
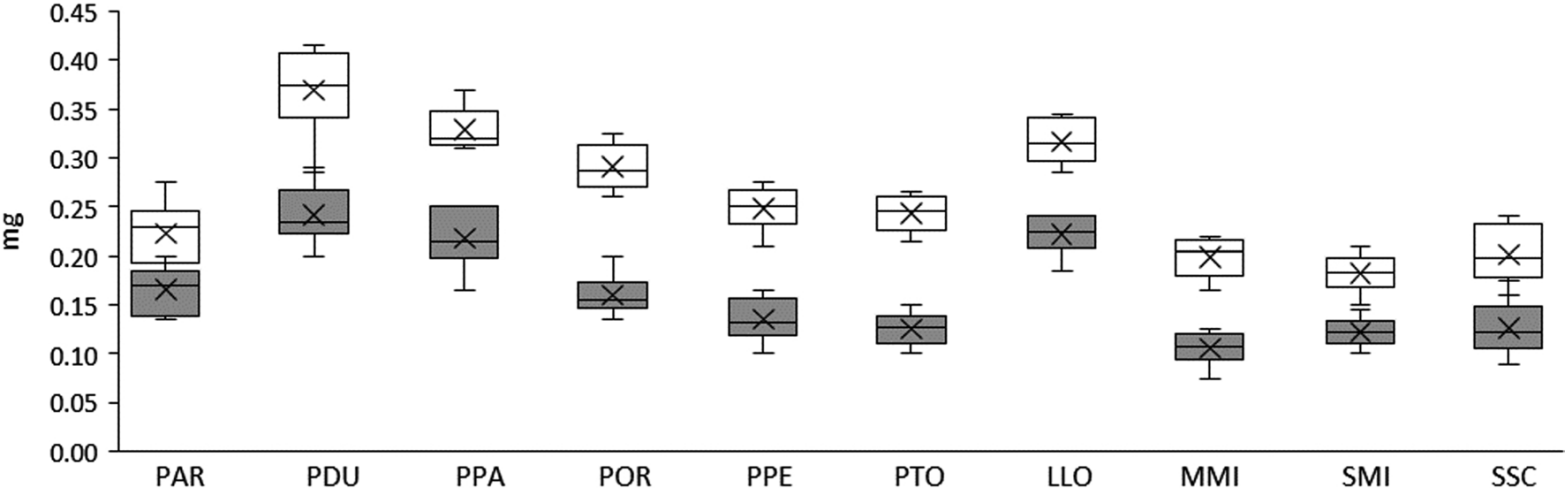
Comparison of body weight of sand fly females and males. The mean unfed body weight of females (white boxes) and males (grey boxes) was measured in ten laboratory-reared sand fly species: *Phlebotomus argentipes* (PAR); *P. duboscqi* (PDU); *P. papatasi* (PPA); *P. orientalis* (POR); *P. perniciosus* (PPE); *P. tobbi* (PTO); *Lutzomyia longipalpis* (LLO); *Migonemyia migonei* (MMI); *Sergentomyia minuta (*SMI) and *S. schwetzi* (SSC). The data represent an average from six independent trials (each sample comprising 20 sand fly specimens). The interspecific differences analysed by one-way ANOVA were highly significant in both females (*F*_*(9,50)*_ = 30.40; *P* < 0.0001) and males (*F*_*(9,50)*_ = 24.49; *P* < 0.0001)

Mean unfed body weights were measured in ten sand fly species. The interspecific comparison revealed highly significant differences in both male and female body weights (see Fig. 2) and in M_m_/M_f_ ratios *(*ANOVA*, F*_*(9,50)*_ = 8.21, *P* < *0.0001*). In *Phlebotomus* species, differences were smaller among members of the same subgenus (*Phlebotomus* and *Larroussius*, respectively); see Table 1.

**Table 1:** Mean unfed body weight of females and males of ten sand fly species

Species	**M**_**f**_ (min–max)	**M**_**m**_ (min–max)	Weight ratio M_m_/M_f_ (SD)	Significance of differences between M_m_ and M_f_
	(mg)	(mg)		
*Phlebotomus argentipes*	**0.223** (0.160–0.275)	**0.166** (0.135–0.200)	**0.75** (0.074)	0.013
*P. duboscqi*	**0.369** (0.285–0.415)	**0.242** (0.200–0.290)	**0.66** (0.071)	0.002
*P. papatasi*	**0.329** (0.310–0.370)	**0.218** (0.165–0.250)	**0.66** (0.082)	< 0.0001
*P. orientalis*	**0.291** (0.260–0.325)	**0.160** (0.135–0.200)	**0.55** (0.069)	< 0.0001
*P. perniciosus*	**0.248** (0**.**210–0.275)	**0.135** (0.100–0.165)	**0.54** (0.072)	< 0.0001
*P. tobbi*	**0.243** (0.215–0.265)	**0.126** (0.100–0.150)	**0.52** (0.059)	< 0.0001
*Lutzomyia longipalpis*	**0.317** (0.285–0.345)	**0.222** (0.185–0.240)	**0.70** (0.065)	< 0.0001
*Migonemyia migonei*	**0.199** (0.165–0.220)	**0.106** (0.075–0.125)	**0.53** (0.082)	< 0.0001
*Sergentomyia minuta*	**0.183** (0.150–0.210)	**0.123** (0.100–0.145)	**0.67** (0.025)	0.0002
*S. schwetzi*	**0.202** (0.160–0.240)	**0.127** (0.090–0.175)	**0.64** (0.073)	0.001

The data represent an average from six independent samples (each comprising 20 sand fly individuals)

Statistical significance of intraspecific differences between males and females was obtained by Student’s t-test

In all species studied, the weight of males was significantly lower than that of females. The smallest difference between sexes was observed in *P. argentipes* and *L. longipalpis* where male weight was about 75% and 70% that of females, respectively. By contrast, in *P. tobbi* and *M. migonei* male weight was only about one half that of females (52% and 53%, respectively); see Fig. 2 and Table 1.

Members of the subgenus *Phlebotomus* were the biggest species studied*: P. duboscqi* (M_f_ 0.369 ± 0.046 mg; M_m_ 0.242 ± 0.031 mg) and *P. papatasi* (M_f_ 0.329 ± 0.022 mg; M_m_ 0.218 ± 0.032 mg). Members of subgenus *Larroussius* ranked among the middle-sized species: *P. orientalis* (M_f_ 0.291 ± 0.025 mg; M_m_ 0.160 ± 0.022 mg), *P. perniciosus* (M_f_ 0.248 ± 0.023 mg; M_m_ 0.135 ± 0.023 mg), and *P. tobbi* (M_f_ 0.243 ± 0.019 mg; M_m_ 0.126 ± 0.017 mg). Females of *P. (Euphlebotomus) argentipes* (M_f_ 0.223 ± 0.038 mg) were the smallest ones in the genus *Phlebotomus* while the males (M_m_ 166 ± 0.025 mg) were slightly heavier than the males of all *Larroussius* species (Fig. 2).

Two species of the genus *Sergentomyia*, *S. minuta* (M_f_ 0.183 ± 0.02 mg; M_m_ 0.123 ± 0.015 mg) and *S. schwetzi* (M_f_ 0.202 ± 0.029 mg; M_m_ 0.127 ± 0.029 mg), did not exhibit any significant difference in either M_f_ and M_m_ or M_m_/M_f_ ratio. On the other hand, considerable differences were found between two New World species studied: *L. longipalpis* values (M_f_ 0.317 ± 0.023 mg and M_m_ 0.222 ± 0.021 mg) ranged close to the values of *P. papatasi*. By contrast, *M. migonei* grouped among the lowest values measured (M_f_ 0.199 ± 0.021 mg and M_m_ 0.106 ± 0.018 mg) and males of *M. migonei* were the smallest males in the study. All data are summarized in Table 1.

### Blood meal size versus body weight of females

M_bl_/M_f_ ratios ranged from 2.25 to 3.05 in most species, with the exception of *P. argentipes* and both *Sergentomyia* species. The females of *P. argentipes*, the tiniest species of the genus *Phlebotomus*, were able to acquire more blood (V_bl_ 0.72 ± 0.06 µl) than females of three relatively bigger species of the *Larroussius* subgenus. If related to their unfed weight (M_bl_/M_f_ 3.34 ± 0.14), they took relatively more blood than the females of all other *Phlebotomus* species tested. Within the subgenus *Larroussius*, the relative consumption was significantly higher in *P. perniciosus* than in the larger females of *P. orientalis* (*P* = 0.015, 95% CI = [0.064, 0.871]) (see S2).

The highest M_bl_/M_f_ ratio was found in both *Sergentomyia* species: 5.18 and 4.55 for *S. minuta* and *S. schwetzi*, respectively. Females of the tiniest species, *S. minuta* (V_bl_ 1.01 ± 0.03 µl) and *S. schwetzi* (V_bl_ 0.96 ± 0.07 µl), ingested higher volumes than the largest species studied, *P. duboscqi* (V_bl_ 0.89 ± 0.06 µl) and *P. papatasi* (V_bl_ 0.90 ± 0.04 µl). Results are summarized in Fig. 1B and Table 2. The Tukey-Kramer multiple comparison test table for M_bl_/M_f_ ratio is provided as “Supplementary information” (Additional file 2: Table S2).

**Table 2:** Blood meal size versus body weight of females

Species	Trial	M_bl_ (mg/gut)	M_f_ (mg/female)	M_bl_/M_f_	Blood meal source
*Phlebotomus argentipes*	I	0.82	0.245	3.37	Mice
	II	0.71	0.205	3.46	
	III	0.72	0.225	3.20	
	Mean	**0.75 ± 0.07**	**0.225 ± 0.02**	**3.34 ± 0.14**	
*P. duboscqi*	I	0.91	0.375	2.42	Mice
	II	1.01	0.415	2.43	
	III	0.90	0.360	2.50	
	Mean	**0.94 ± 0.06**	**0.383 ± 0.03**	**2,45 ± 0.04**	
*P. papatasi*	I	0.96	0.365	2.64	Mice
	II	0.98	0.375	2.62	
	III	0.90	0.320	2.80	
	Mean	**0.95 ± 0.05**	**0.353 ± 0.03**	**2.69 ± 0.10**	
*P. orientalis*	I	0.46	0.200	2.29	Rabbit
	II	0.58	0.260	2.22	
	III	0.61	0.275	2.23	
	Mean	**0.55 ± 0.08**	**0.245 ± 0.04**	**2.25 ± 0.04**	
*P. perniciosus*	I	0.72	0.275	2.63	Rabbit
	II	0.69	0.265	2.60	
	III	0.61	0.210	2.91	
	Mean	**0.68 ± 0.06**	**0.250 ± 0.04**	**2.72 ± 0.17**	
*P. tobbi*	I	0.58	0.250	2.32	Rabbit
	II	0.54	0.215	2.50	
	III	0.61	0.260	2.36	
	Mean	**0.58 ± 0.04**	**0.242 ± 0.02**	**2.40 ± 0.10**	
*Lutzomyia longipalpis*	I	0.91	0.290	3.12	Mice
	II	0.88	0.285	3.07	
	III	1.02	0.345	2.94	
	Mean	**0.93 ± 0.07**	**0.307 ± 0.03**	**3.05 ± 0.09**	
*Migonemyia migonei*	I	0.50	0.220	2.25	Rabbit
	II	0.53	0.225	2.36	
	III	0.51	0.200	2.57	
	Mean	**0.51 ± 0.02**	**0.215 ± 0.01**	**2.39 ± 0.16**	
*Sergentomyia minuta*	I	0.99	0.185	5.37	Gecko
	II	1.06	0.210	5.03	
	III	1.00	0.195	5.15	
	Mean	**1.02 ± 0.03**	**0.197 ± 0.01**	**5.18 ± 0.17**	
*S. schwetzi*	I	0.94	0.195	4.81	Mice
	II	1.08	0.240	4.51	
	III	0.99	0.230	4.32	
	Mean	**1.00 ± 0.07**	**0.222 ± 0.02**	**4.55 ± 0.25**	

The mean blood meal weight (M_bl_) was determined and related to the mean body weight of an unfed female (M_f_)

## Discussion

In ten sand fly species studied, the mean volume of ingested blood meal ranged from 0.47 to 1.01 µl. This size is higher than that in biting midges (Ceratopogonidae) but smaller than in mosquitoes (Culicidae). In biting midges, the mean blood meal size was 0.44 mg for *Culicoides* *variipennis* (by ELISA test) [17] and 0.36 mg for *Culicoides arakawae* (by chemical analyses) [23]. For *Aedes aegypti*, blood intake has been reported by several studies (reviewed in [24]). For instance, Woke et al. [25] determined the blood meal range from 1.5 to 3.9 mg by gravimetry, while Briegel quantified [20] blood meals using excretory haematin measurement, finding a range of 1.3 to 6.6 µl.

In most sand fly species studied (eight *Phlebotomus* and *Lutzomyia* species), the relation of blood meal size to the size of females (relative consumption) was similar to that ofmosquitoes but higher than that in biting midges. If fed to repletion, mosquito females can ingest blood meals 2–4 times their body weight [26], while *C. variipennis* females fed on horse blood retained 1.2–1.9 times their unfed weight in blood [17]. In all sand fly species tested, the largest blood intake was documented in females from cohorts with the highest mean weight. However, the highest relative consumption (Mbl/Mf ratio) was observed in cohorts with the lowest weight in most species tested. No positive correlation was found between mean blood volume and mean size of sand fly females by interspecific comparison.

Very high relative consumption was found in both *Sergentomyia* species studied; they ingested blood meals 4.5–5 times greater than their body weight. This high blood consumption (both absolute and relative) affirms the large volumes reported by previous studies: 0.91 µl on anaesthetized mice and 0.82 µl by artificial feeding for *S. schwetzi* and 0.97 µl on human arms and 1.02 µl on geckos for *S. minuta* [7, 15]. While *S. minuta* is mainly herpetophilic [15], *S. schwetzi* is an opportunistic feeder [27] that readily feeds on reptiles; a colony of *S. schwetzi* fed solely on geckos was successfully maintained in the laboratory for 8 years [28]. The substantial amounts of blood acquired may reflect an adaptation of *Sergentomyia* species to the lower haemoglobin content in reptilian erythrocytes [29], requiring a large gut capacity and highly efficient blood meal concentration during feeding. Additionally, *S. minuta* feeds on geckos for up to 45 min to full repletion, enabled by the lack of defensive behaviour in reptiles [15]. In mosquitoes, a similarly long feeding time of up to 40 min has been observed in *Culex territans*, which primarily feeds on cold-blooded vertebrates [30]. In contrast, the low consumption observed in *M. migonei* may reflect its ornithophilic feeding preferences [31], where a fast feeding strategy reduces the risk of active defensive behaviour by birds.

Among *Phlebotomus* species, the tiny females of *P. argentipes* showed the highest relative consumption. Our results align with previous findings by Pruzinova et al. [7] which reported blood meal volumes of 0.73 µl on anaesthetized mice and 0.63 µl on rabbit blood via a chick-skin membrane. All three *Larroussius* species studied (*P. perniciosus*, *P. orientalis*, and *P. tobbi*) took similar blood volumes while feeding on rabbits (mean Vbl = 0.51–0.61 µl). However, *P. perniciosus* females showed the highest relative consumption, possibly because they readily feed on hares and wild rabbits [32], whereas *P. orientalis* and *P. tobbi* prefer large livestock and humans [33, 34]. Similar volumes were previously described for *P. orientalis* feeding on mice or through a membrane on rabbit blood (0.53 µl and 0.59 µl) [7]. For *Phlebotomus (Larroussius) langeroni* membrane fed on defibrinated human blood, larger blood meals were observed (0.76–0.94 µl and 0.71–0.99 µl, measured by protein content and haemoglobin methods, respectively) [13].

The blood meal volume of 0.89 µl reported here for *L. longipalpis* is higher than volumes estimated previously for this species by gravimetry: 0.55 mg (maximum 0.75 mg [12]. Similar differences, caused by different methodologies, were observed in *P. papatasi*. Here, the mean blood volume was 0.90 µl, matching previous findings in *P. papatasi* females feeding on anaesthetized mice and measured by haemoglobinometry [7]. In contrast, Theodor [10], using gravimetry for *P. papatasi*, determined a mean blood meal weight of 0.4–0.5 mg (maximum 0.58 mg). These differences are due to prediuresis: excretion of excessive water and concentration of the blood meal during feeding. Prediuretic excretion is a physiological mechanism used by haematophagous arthropods to control water balance and body temperature and to concentrate the blood meal during feeding [35, 36]. Diuresis, initiated after feeding, reduces the flight weight of the freshly fed female [16]. Very efficient prediuresis was described in *Anopheles* mosquitoes, where *Anopheles stephensi* can have a maximum gut capacity of 2–3 µl but mean blood meal consumption can reach 6 µl because of extensive prediuretic excretion [16, 37].

In sand flies, prediuretic excretion was previously documented in 100% of *P. argentipes*[18], 100% of *P. papatasi*, and 85% of *P. duboscqi* females [19] and in the majority of *L. longipalpis* females [3]. Variations in urine production correlated with the length of feeding, with *P. papatasi* having a significantly longer excretion time and producing more droplets than *P. duboscqi* [19]. These interspecific discrepancies in prediuresis patterns correspond with the higher Mbl/Mf ratio of *P. papatasi* compared to *P. duboscqi* documented in this study.

Analogously to blood meal size, the mean weight of sand fly females also ranged between the largest biting midges (e.g. 0.2501 ± 0.0587 mg of *Culicoides variipennis*) [17] and small mosquito species (e.g. 0.7 ± 0.1 mg of *Anopheles minimus*) [21]. Similarly to other Nematocera, the body size of sand flies has mostly been determined using morphometric parameters. The only data on their body weight came from studies of Israeli populations of *P. papatasi*. Adler and Theodor [38] reported an average female weight of 0.3 mg. The unfed weight of males and females caught in Jerusalem was 0.24–0.28 mg and 0.35–0.4 mg, respectively [10]. Population differences were described by Jacobson et al. [22] among four *P. papatasi* colonies from diverse ecological habitats and seasons. The mean unfed weight (48 h after emergence) was 295 µg (95% CI 0.277–0.312) in females and 223 µg (95% CI 0.213–0.233) in males. Oasis flies were smaller than desert flies, and the autumn line of flies from super arid areas was significantly heavier than for flies from other localities [22].

This study was conducted under standard laboratory conditions. However, in natural environments, we expect higher variability in the blood meal volumes taken by sand fly females, likely due to the diversity of hosts and their defensive behaviours. Furthermore, some sand fly species or populations are known to be gonotrophically discordant. For example, in various populations of *P. papatasi*, females have been repeatedly observed taking multiple blood meals (2–4) within a single gonotrophic cycle [10, 39]. The implications of this behaviour for the transmission of human pathogens have been extensivelydiscussed for *P. papatasi* and *P. duboscqi* [40].

## Conclusions

Sand fly species significantly differ in blood meal volume taken by females under standard conditions. Five species from three genera (*P. papatasi, P. duboscqi, L. longipalpis, S. minuta, and S. schwetzi*) took double the blood meal compared to *M. migonei*. These intraspecific differences are crucial for determining optimal pathogen doses (e.g. *Leishmania* or phleboviruses) during experimental infections, such as comparative studies with New World species *L. longipalpis* and *M. migonei* [41]. The relation of blood meal amount to female size (relative consumption) had not been studied in sand flies before to our knowledge. Interestingly, in all *Phlebotomus* and *Lutzomyia* species studied, the Mbl/Mf ratio ranged between 2.25 and 3.34. In contrast, both *Sergentomyia* species studied ingested blood meals 4.5–5 times their body weight. For future research, we recommend testing the blood meal volumes taken by sand fly females under natural conditions. Haemoglobinometry would be an optimal assay for such a study.

### Supplementary Information

Additional file 1.

Additional file 2.

## Data Availability

Data are provided within the manuscript or supplementary information files.
