# Supplementary material for: Sand fly blood meal volumes and their relation to female body weight under experimental conditions
Source: Parasit Vectors. 2024 Aug 23;17:360. doi: 10.1186/s13071-024-06418-y (PMC11342606; doi:10.1186/s13071-024-06418-y)
Supplement: Supplementary file 1 — Additional file 1. [file 13071_2024_6418_MOESM1_ESM.docx]

**Supplementary information**

**Additional file1: Table S1.** Tukey HSD test for blood meal volume

|  | TUKEY HSD/KRAMER | | | |  | | alpha | 0,05 |  |
| --- | --- | --- | --- | --- | --- | --- | --- | --- | --- |
| *Species* | | *group* | | *mean* | | *n* | *ss* | *df* | *q-crit* |
| *P. papatasi* | | PPA | | 0,90202 | | 3 | 0,003632 |  |  |
| *P. duboscqi* | | PDU | | 0,894666 | | 3 | 0,006773 |  |  |
| *P. orientalis* | | POR | | 0,514869 | | 3 | 0,009131 |  |  |
| *P. perniciosus* | | PPE | | 0,61377 | | 3 | 0,005503 |  |  |
| *P. tobbi* | | PTO | | 0,541262 | | 3 | 0,001251 |  |  |
| *P. argentipes* | | PAR | | 0,715725 | | 3 | 0,007562 |  |  |
| *L. longipalpis* | | LLO | | 0,887731 | | 3 | 0,009834 |  |  |
| *M. migonei* | | MMI | | 0,466534 | | 3 | 0,000473 |  |  |
| *S. schwetzi* | | SSC | | 0,956779 | | 3 | 0,009641 |  |  |
| *S. minuta* | | SMI | | 1,00778 | | 3 | 0,002231 |  |  |
|  |  | |  | | | 30 | 0,056031 | 20 | 5,008 |

| Q TEST |  |  |  |  |  |  |  |  |  |
| --- | --- | --- | --- | --- | --- | --- | --- | --- | --- |
| *group 1* | *group 2* | *mean* | *std err* | *q-stat* | *lower* | *upper* | *p-value* | *mean-crit* | *Cohen d* |
| PPA | PDU | 0,007355 | 0,030559 | 0,240667 | -0,14568 | 0,160394 | 1 | 0,153039 | 0,138949 |
| PPA | POR | 0,387151 | 0,030559 | 12,66896 | 0,234111 | 0,54019 | 7,38E-07 | 0,153039 | 7,314429 |
| PPA | PPE | 0,28825 | 0,030559 | 9,432579 | 0,135211 | 0,44129 | 6,11E-05 | 0,153039 | 5,445902 |
| PPA | PTO | 0,360758 | 0,030559 | 11,8053 | 0,207719 | 0,513798 | 2,25E-06 | 0,153039 | 6,815794 |
| PPA | PAR | 0,186295 | 0,030559 | 6,096246 | 0,033256 | 0,339335 | 0,009851 | 0,153039 | 3,51967 |
| PPA | LLO | 0,01429 | 0,030559 | 0,467607 | -0,13875 | 0,167329 | 0,999998 | 0,153039 | 0,269973 |
| PPA | MMI | 0,435487 | 0,030559 | 14,25068 | 0,282447 | 0,588526 | 1,07E-07 | 0,153039 | 8,227634 |
| PPA | SSC | 0,054759 | 0,030559 | 1,791904 | -0,09828 | 0,207798 | 0,950174 | 0,153039 | 1,034556 |
| PPA | SMI | 0,10576 | 0,030559 | 3,46085 | -0,04728 | 0,2588 | 0,350856 | 0,153039 | 1,998123 |
| PDU | POR | 0,379796 | 0,030559 | 12,4283 | 0,226757 | 0,532836 | 1E-06 | 0,153039 | 7,17548 |
| PDU | PPE | 0,280896 | 0,030559 | 9,191913 | 0,127856 | 0,433935 | 8,7E-05 | 0,153039 | 5,306953 |
| PDU | PTO | 0,353404 | 0,030559 | 11,56463 | 0,200364 | 0,506443 | 3,1E-06 | 0,153039 | 6,676845 |
| PDU | PAR | 0,178941 | 0,030559 | 5,85558 | 0,025901 | 0,33198 | 0,014226 | 0,153039 | 3,380721 |
| PDU | LLO | 0,006935 | 0,030559 | 0,22694 | -0,1461 | 0,159975 | 1 | 0,153039 | 0,131024 |
| PDU | MMI | 0,428132 | 0,030559 | 14,01001 | 0,275093 | 0,581171 | 1,43E-07 | 0,153039 | 8,088685 |
| PDU | SSC | 0,062113 | 0,030559 | 2,032571 | -0,09093 | 0,215153 | 0,900761 | 0,153039 | 1,173505 |
| PDU | SMI | 0,113115 | 0,030559 | 3,701517 | -0,03992 | 0,266154 | 0,271605 | 0,153039 | 2,137072 |
| POR | PPE | 0,098901 | 0,030559 | 3,236384 | -0,05414 | 0,25194 | 0,435933 | 0,153039 | 1,868527 |
| POR | PTO | 0,026393 | 0,030559 | 0,863663 | -0,12665 | 0,179432 | 0,999728 | 0,153039 | 0,498636 |
| POR | PAR | 0,200856 | 0,030559 | 6,572717 | 0,047816 | 0,353895 | 0,004727 | 0,153039 | 3,79476 |
| POR | LLO | 0,372861 | 0,030559 | 12,20136 | 0,219822 | 0,525901 | 1,34E-06 | 0,153039 | 7,044456 |
| POR | MMI | 0,048336 | 0,030559 | 1,581716 | -0,1047 | 0,201375 | 0,976672 | 0,153039 | 0,913204 |
| POR | SSC | 0,44191 | 0,030559 | 14,46087 | 0,28887 | 0,594949 | 8,39E-08 | 0,153039 | 8,348986 |
| POR | SMI | 0,492911 | 0,030559 | 16,12981 | 0,339872 | 0,64595 | 1,3E-08 | 0,153039 | 9,312552 |
| PPE | PTO | 0,072508 | 0,030559 | 2,372722 | -0,08053 | 0,225547 | 0,794996 | 0,153039 | 1,369891 |
| PPE | PAR | 0,101955 | 0,030559 | 3,336333 | -0,05108 | 0,254994 | 0,39685 | 0,153039 | 1,926233 |
| PPE | LLO | 0,273961 | 0,030559 | 8,964972 | 0,120921 | 0,427 | 0,000122 | 0,153039 | 5,175929 |
| PPE | MMI | 0,147236 | 0,030559 | 4,8181 | -0,0058 | 0,300276 | 0,065448 | 0,153039 | 2,781732 |
| PPE | SSC | 0,343009 | 0,030559 | 11,22448 | 0,18997 | 0,496048 | 4,9E-06 | 0,153039 | 6,480459 |
| PPE | SMI | 0,39401 | 0,030559 | 12,89343 | 0,240971 | 0,54705 | 5,57E-07 | 0,153039 | 7,444025 |
| PTO | PAR | 0,174463 | 0,030559 | 5,709054 | 0,021424 | 0,327502 | 0,017763 | 0,153039 | 3,296124 |
| PTO | LLO | 0,346469 | 0,030559 | 11,33769 | 0,193429 | 0,499508 | 4,2E-06 | 0,153039 | 6,545821 |
| PTO | MMI | 0,074728 | 0,030559 | 2,445379 | -0,07831 | 0,227768 | 0,767745 | 0,153039 | 1,41184 |
| PTO | SSC | 0,415517 | 0,030559 | 13,5972 | 0,262478 | 0,568556 | 2,34E-07 | 0,153039 | 7,85035 |
| PTO | SMI | 0,466518 | 0,030559 | 15,26615 | 0,313479 | 0,619558 | 3,35E-08 | 0,153039 | 8,813917 |
| PAR | LLO | 0,172006 | 0,030559 | 5,62864 | 0,018966 | 0,325045 | 0,020051 | 0,153039 | 3,249697 |
| PAR | MMI | 0,249191 | 0,030559 | 8,154433 | 0,096152 | 0,402231 | 0,000412 | 0,153039 | 4,707964 |
| PAR | SSC | 0,241054 | 0,030559 | 7,888151 | 0,088015 | 0,394093 | 0,000619 | 0,153039 | 4,554226 |
| PAR | SMI | 0,292055 | 0,030559 | 9,557097 | 0,139016 | 0,445095 | 5,1E-05 | 0,153039 | 5,517792 |
| LLO | MMI | 0,421197 | 0,030559 | 13,78307 | 0,268157 | 0,574236 | 1,87E-07 | 0,153039 | 7,957661 |
| LLO | SSC | 0,069048 | 0,030559 | 2,259511 | -0,08399 | 0,222088 | 0,834497 | 0,153039 | 1,304529 |
| LLO | SMI | 0,12005 | 0,030559 | 3,928457 | -0,03299 | 0,273089 | 0,209231 | 0,153039 | 2,268096 |
| MMI | SSC | 0,490245 | 0,030559 | 16,04258 | 0,337206 | 0,643285 | 1,42E-08 | 0,153039 | 9,26219 |
| MMI | SMI | 0,541247 | 0,030559 | 17,71153 | 0,388207 | 0,694286 | 2,5E-09 | 0,153039 | 10,22576 |
| SSC | SMI | 0,051001 | 0,030559 | 1,668946 | -0,10204 | 0,204041 | 0,967381 | 0,153039 | 0,963567 |
