# Supplementary material for: Sand fly blood meal volumes and their relation to female body weight under experimental conditions
Source: Parasit Vectors. 2024 Aug 23;17:360. doi: 10.1186/s13071-024-06418-y (PMC11342606; doi:10.1186/s13071-024-06418-y)
Supplement: Supplementary file 2 — Additional file 2. [file 13071_2024_6418_MOESM2_ESM.docx]

**Supplementary information**

**Additional file2: Table S2.** Tukey HSD test for M_bl_/M_f_ ratio

|  | TUKEY HSD/KRAMER | | | |  | | alpha | 0,05 |  |
| --- | --- | --- | --- | --- | --- | --- | --- | --- | --- |
| *Species* | | *group* | | *mean* | | *n* | *ss* | *df* | *q-crit* |
| *P. papatasi* | | PPA | | 2,686091 | | 3 | 0,020342 |  |  |
| *P. duboscqi* | | PDU | | 2,452047 | | 3 | 0,003897 |  |  |
| *P. orientalis* | | POR | | 2,248611 | | 3 | 0,003234 |  |  |
| *P. perniciosus* | | PPE | | 2,716114 | | 3 | 0,058753 |  |  |
| *P. tobbi* | | PTO | | 2,39585 | | 3 | 0,018184 |  |  |
| *P. argentipes* | | PAR | | 3,342737 | | 3 | 0,03651 |  |  |
| *L. longipalpis* | | LLO | | 3,045791 | | 3 | 0,016967 |  |  |
| *M. migonei* | | MMI | | 2,392653 | | 3 | 0,051276 |  |  |
| *S. schwetzi* | | SSC | | 4,546467 | | 3 | 0,120936 |  |  |
| *S. minuta* | | SMI | | 5,148379 | | 3 | 0,05899 |  |  |
|  |  | |  | | | 30 | 0,389089 | 20 | 5,008 |

| Q TEST |  |  |  |  |  |  |  |  |  |
| --- | --- | --- | --- | --- | --- | --- | --- | --- | --- |
| *group 1* | *group 2* | *mean* | *std err* | *q-stat* | *lower* | *upper* | *p-value* | *mean-crit* | *Cohen d* |
| PPA | PDU | 0,234044 | 0,080528 | 2,906354 | -0,16924 | 0,63733 | 0,57463 | 0,403286 | 1,677984 |
| PPA | POR | 0,43748 | 0,080528 | 5,432622 | 0,034194 | 0,840767 | 0,026879 | 0,403286 | 3,136526 |
| PPA | PPE | 0,030023 | 0,080528 | 0,372825 | -0,37326 | 0,433309 | 1 | 0,403286 | 0,215251 |
| PPA | PTO | 0,290241 | 0,080528 | 3,604207 | -0,11305 | 0,693527 | 0,302048 | 0,403286 | 2,08089 |
| PPA | PAR | 0,656645 | 0,080528 | 8,154208 | 0,253359 | 1,059932 | 0,000412 | 0,403286 | 4,707834 |
| PPA | LLO | 0,359699 | 0,080528 | 4,466739 | -0,04359 | 0,762986 | 0,105905 | 0,403286 | 2,578873 |
| PPA | MMI | 0,293439 | 0,080528 | 3,643915 | -0,10985 | 0,696725 | 0,289358 | 0,403286 | 2,103815 |
| PPA | SSC | 1,860375 | 0,080528 | 23,1021 | 1,457089 | 2,263662 | 1,96E-11 | 0,403286 | 13,338 |
| PPA | SMI | 2,462288 | 0,080528 | 30,57664 | 2,059002 | 2,865574 | 1,13E-13 | 0,403286 | 17,65343 |
| PDU | POR | 0,203436 | 0,080528 | 2,526268 | -0,19985 | 0,606723 | 0,735936 | 0,403286 | 1,458542 |
| PDU | PPE | 0,264067 | 0,080528 | 3,279179 | -0,13922 | 0,667353 | 0,418981 | 0,403286 | 1,893235 |
| PDU | PTO | 0,056197 | 0,080528 | 0,697853 | -0,34709 | 0,459483 | 0,999953 | 0,403286 | 0,402906 |
| PDU | PAR | 0,890689 | 0,080528 | 11,06056 | 0,487403 | 1,293976 | 6,12E-06 | 0,403286 | 6,385818 |
| PDU | LLO | 0,593743 | 0,080528 | 7,373092 | 0,190457 | 0,99703 | 0,001368 | 0,403286 | 4,256857 |
| PDU | MMI | 0,059395 | 0,080528 | 0,737562 | -0,34389 | 0,462681 | 0,999926 | 0,403286 | 0,425831 |
| PDU | SSC | 2,094419 | 0,080528 | 26,00846 | 1,691133 | 2,497706 | 2,18E-12 | 0,403286 | 15,01599 |
| PDU | SMI | 2,696332 | 0,080528 | 33,48299 | 2,293046 | 3,099618 | 3,03E-14 | 0,403286 | 19,33142 |
| POR | PPE | 0,467503 | 0,080528 | 5,805447 | 0,064217 | 0,87079 | 0,015352 | 0,403286 | 3,351776 |
| POR | PTO | 0,147239 | 0,080528 | 1,828415 | -0,25605 | 0,550526 | 0,944058 | 0,403286 | 1,055636 |
| POR | PAR | 1,094126 | 0,080528 | 13,58683 | 0,690839 | 1,497412 | 2,37E-07 | 0,403286 | 7,84436 |
| POR | LLO | 0,79718 | 0,080528 | 9,899361 | 0,393893 | 1,200466 | 3,11E-05 | 0,403286 | 5,715398 |
| POR | MMI | 0,144042 | 0,080528 | 1,788706 | -0,25924 | 0,547328 | 0,950687 | 0,403286 | 1,03271 |
| POR | SSC | 2,297856 | 0,080528 | 28,53472 | 1,894569 | 2,701142 | 3,97E-13 | 0,403286 | 16,47453 |
| POR | SMI | 2,899768 | 0,080528 | 36,00926 | 2,496482 | 3,303055 | 1,88E-14 | 0,403286 | 20,78996 |
| PPE | PTO | 0,320264 | 0,080528 | 3,977032 | -0,08302 | 0,72355 | 0,197427 | 0,403286 | 2,29614 |
| PPE | PAR | 0,626622 | 0,080528 | 7,781383 | 0,223336 | 1,029909 | 0,000729 | 0,403286 | 4,492584 |
| PPE | LLO | 0,329676 | 0,080528 | 4,093914 | -0,07361 | 0,732963 | 0,171174 | 0,403286 | 2,363622 |
| PPE | MMI | 0,323462 | 0,080528 | 4,01674 | -0,07982 | 0,726748 | 0,188172 | 0,403286 | 2,319066 |
| PPE | SSC | 1,830352 | 0,080528 | 22,72928 | 1,427066 | 2,233639 | 2,65E-11 | 0,403286 | 13,12275 |
| PPE | SMI | 2,432265 | 0,080528 | 30,20382 | 2,028979 | 2,835551 | 1,41E-13 | 0,403286 | 17,43818 |
| PTO | PAR | 0,946886 | 0,080528 | 11,75841 | 0,5436 | 1,350173 | 2,4E-06 | 0,403286 | 6,788724 |
| PTO | LLO | 0,64994 | 0,080528 | 8,070946 | 0,246654 | 1,053227 | 0,000468 | 0,403286 | 4,659763 |
| PTO | MMI | 0,003198 | 0,080528 | 0,039709 | -0,40009 | 0,406484 | 1 | 0,403286 | 0,022926 |
| PTO | SSC | 2,150616 | 0,080528 | 26,70631 | 1,74733 | 2,553903 | 1,34E-12 | 0,403286 | 15,41889 |
| PTO | SMI | 2,752529 | 0,080528 | 34,18085 | 2,349243 | 3,155815 | 2,5E-14 | 0,403286 | 19,73432 |
| PAR | LLO | 0,296946 | 0,080528 | 3,687469 | -0,10634 | 0,700232 | 0,275863 | 0,403286 | 2,128961 |
| PAR | MMI | 0,950084 | 0,080528 | 11,79812 | 0,546798 | 1,35337 | 2,28E-06 | 0,403286 | 6,81165 |
| PAR | SSC | 1,20373 | 0,080528 | 14,94789 | 0,800444 | 1,607016 | 4,79E-08 | 0,403286 | 8,630171 |
| PAR | SMI | 1,805643 | 0,080528 | 22,42243 | 1,402356 | 2,208929 | 3,42E-11 | 0,403286 | 12,9456 |
| LLO | MMI | 0,653138 | 0,080528 | 8,110654 | 0,249852 | 1,056424 | 0,000441 | 0,403286 | 4,682688 |
| LLO | SSC | 1,500676 | 0,080528 | 18,63536 | 1,09739 | 1,903962 | 1,01E-09 | 0,403286 | 10,75913 |
| LLO | SMI | 2,102589 | 0,080528 | 26,1099 | 1,699302 | 2,505875 | 2,03E-12 | 0,403286 | 15,07456 |
| MMI | SSC | 2,153814 | 0,080528 | 26,74602 | 1,750528 | 2,5571 | 1,31E-12 | 0,403286 | 15,44182 |
| MMI | SMI | 2,755727 | 0,080528 | 34,22056 | 2,35244 | 3,159013 | 2,48E-14 | 0,403286 | 19,75725 |
| SSC | SMI | 0,601913 | 0,080528 | 7,474539 | 0,198626 | 1,005199 | 0,00117 | 0,403286 | 4,315427 |
